# Supplementary material for: Retrospective study of a novel hematological parameter for predicting the survival of patients with nasopharyngeal carcinoma
Source: PeerJ. 2024 Jun 21;12:e17573. doi: 10.7717/peerj.17573 (PMC11195549; doi:10.7717/peerj.17573)
Supplement: Supplemental Information 3 — Supplement to the original data [file peerj-12-17573-s003.docx]

AJCC 7th edition:
T
T1 - confined to nasopharynx, or tumor extends to oropharynx and/or nasal cavity without parapharyngeal extension

T2 - tumor with para pharyngeal extension (posterolateral infiltration of tumor, i.e. beyond the pharyngobasilar fascia)
T3 - involves bony structures and/or paranasal sinuses
T4- intracranial extension and/or involvement of cranial nerves, infratemporal fossa, hypopharynx, or bit, or masticator space Nodes
N
N1 - unilateral nodes, 6 cm or less, above the supraclavicular fossa, and/or retropharyngeal lymphnodes 7 cm or less (unilateral or bilateral)
N2 - bilateral nodes, 6 cm or less, above the supraclav fossa
N3a - lymph node greater than 6 cm
N3b - extension to the supraclav fossa (defined as the triangular region described by Ho, bounded by the superior margin of the sternal head of the clavicle, the superior margin of the lateral end of the clavicle, and the point where the neck meets the shoulder. This includes some of level lV as well as V.)

M
M0 -distant metastasis
M1 -no distant metastasis
stage
I:T1 N0
II:T1-T2 N1，T2 N0 (i.e.T2 or N1)
III:T3 N0-2, or T1-3 N2 (i.e.T3 or N2)
IVA:T4 N0-2
IVB:N3
